# Progress of Research on the Application of Nanoelectronic Smelling in the Field of Food

**DOI:** 10.3390/mi13050789

**Published:** 2022-05-18

**Authors:** Junjiang Sha, Chong Xu, Ke Xu

**Affiliations:** School of Electrical & Control Engineering, Shenyang Jianzhu University, Shenyang 110168, China; shajunjiang1013@163.com (J.S.); xksky1234@163.com (K.X.)

**Keywords:** nanoelectronic smelling, food identification, flavor compounds

## Abstract

In the past 20 years, the development of an artificial olfactory system has made great progress and improvements. In recent years, as a new type of sensor, nanoelectronic smelling has been widely used in the food and drug industry because of its advantages of accurate sensitivity and good selectivity. This paper reviews the latest applications and progress of nanoelectronic smelling in animal-, plant-, and microbial-based foods. This includes an analysis of the status of nanoelectronic smelling in animal-based foods, an analysis of its harmful composition in plant-based foods, and an analysis of the microorganism quantity in microbial-based foods. We also conduct a flavor component analysis and an assessment of the advantages of nanoelectronic smelling. On this basis, the principles and structures of nanoelectronic smelling are also analyzed. Finally, the limitations and challenges of nanoelectronic smelling are summarized, and the future development of nanoelectronic smelling is proposed.

## 1. Introduction

Nowadays, increasing demand for food quality requires an efficient, convenient, and reliable method for quality control in the food industry, and nanomaterials and structures have broad development and application prospects in the field of olfactory systems [1]. Nanoelectronic smelling has become one of the most sought-after nanosensors due to its excellent selectivity and precise sensitivity. Unlike conventional gas sensors, nanometer electronic smelling can generate a unique response to certain gases through a sensor array with a pattern recognition system to receive the acquisition of data, simulating the human brain in analyzing smell. Not only that, but nanoelectronic smelling can also be combined with a variety of other methods. For example, machine learning, pattern recognition technology, principal component analysis, nearest Neighbor method (KNN), partial least-squares, and artificial neural networks to build mathematical models, especially in food-related industries, using data visualization to convey clear and efficient information to usersthrough infographics such as charts. This will help users analyze and interpret data and make it easier to access, understand, and use. Its advantages are that specific sensor arrays can be designed according to specific smells, which greatly increases the accuracy of data and makes it more convenient to build corresponding mathematical models for prediction. Traditional gas sensors can only analyze a single gas substance, and if there is interference bias, incorrect data will be obtained. The multi-sensor array of nanoelectronic smelling can carefully analyze all kinds of odorous substances so that the error rate is reduced. In the last two decades, nanoelectronic smelling has been known for its ability to work with metal particles (such as Pt, Pd, RH, Pt-Y, Y, SC, Ag, Au,), 2D nanomaterials (such as MoS_2_, WSe_2_), WO_3_, nanofibers (NFs), chemo resistors, phosphoric acid nanosheets (PNS), single-arm carbon nanotubes (SWNT), and other nanomaterials [2]. Nowadays, the application of nanoelectronic smelling in the food industry has become a research hotspot.

In the field of food identification, to the ability to predict the quality, grade, and crop maturity of food products just by smell has long been a challenge for researchers, and nanoelectronic smelling devices have multiple advantages, including high sensitivity, simplicity, rapidity, small sample requirements, and excellent correlation based on sensory evaluation data. Henike Guilherme Jordan Voss et al. [3] constructed a nanoelectronic smelling model consisting of a sensor array of thirteen metal oxide semiconductor sensors to monitor the growth cycle of peaches. Their nanoelectronic smelling model was used to build an exponential model to describe the quality of peaches at different harvest periods, and the model predicted with an accuracy of 85%. Therefore, this nanoelectronic smelling method may be a good device for peach farmers to use to predict the harvest time of peaches and reduce the losses incurred when peaches are harvested while still immature. Sara Gaggiotti et al. [4] used nanoelectronic smelling based on an array of six quartz crystal microbalances (QCMs) sensors with a modified QCMs sensor using peptide-functionalized zinc oxide nanoparticles to analyze the quality of flour produced by different wheat-processing methods with the main objective of detecting low or high furosine content to carry out the differentiation between pasta samples of different commercial values. This study helps to differentiate between high- and low-priced pasta products in the market and prevent consumers from being defrauded. The simplicity and speed with which the identification can be done without destroying the food product makes nanoelectronic smelling a great alternative for quality control in the food industry. Different foods have different flavors and the analysis of the flavor components that we love is troublesome. The emergence of nanoelectronic smelling not only allows for an analysis of the flavor components of food but also the flavor characteristics of the food, for example, nanoelectronic smelling can analyze the flavor components of cooked meats and smoked sausages or the grade of different varieties of cocoa beans, tea, etc. Lu Wang et al. [5] The study was conducted using gas chromatography-mass spectrometry and nanoelectronic smelling to analyze the volatiles of sugarcane juice. Thirteen volatiles were analyzed and alcohol was found to be the main volatile in sugarcane juice. This study evaluated the flavor of different varieties of sugarcane using nanoelectronic smelling, which provides an effective identification pathway for this industry. Jiahui Zhang et al. [6] analyzed the flavor components of golden pomfret fillets treated by four methods: vacuum freezing, hot air, microwave, and vacuum microwave using nanoelectronic smelling, prepared based on ten oxide semiconductors, and the results showed a total of 86 volatile flavor components. The main flavor components were hydrocarbons, aldehydes, esters, and alcohols, and the hot air, microwave, and vacuum microwave treatment methods resulted in a decrease in ketones and an increase in esters in the fillets, whereas the vacuum-freezing treatment method resulted in more hydrocarbons and alcohols in the fillets, providing a reference for the treatment of golden pomfret. Compared to a dog’s nose, nanoelectronic smelling is more accurate and reliable. It can accurately distinguish the subtle differences in the flavor components of the same foods by different processing methods and can also be combined with various analytical processing methods to process the collected data. For example, Mahdi Ghasemi-Varnamkhasti et al. [7] used nanoelectronic smelling based on a five-odor sensor array combined with principal component analysis, linear discriminant analysis, SVM, partial least-squares, polymerase chain reaction, and artificial neural network methods for the storage of French cheese, laying the foundations for the application of nanoelectronic smelling in combination with other methods. To sum up, the advantages of using nanoelectronic smelling are that it combines good data correlation with high sensitivity that is based on the human sensory panel for specific applications, such as easy to build food monitoring systems, real-time detection, economic efficiency, online monitoring of volatiles, non-destructive technology, and a shorter time required for analysis. The combination of various technologies and nanoelectronic smelling for real-time-related systems has proved to be a reliable approach for aroma and flavor quality assessment.

**Figure 1:**
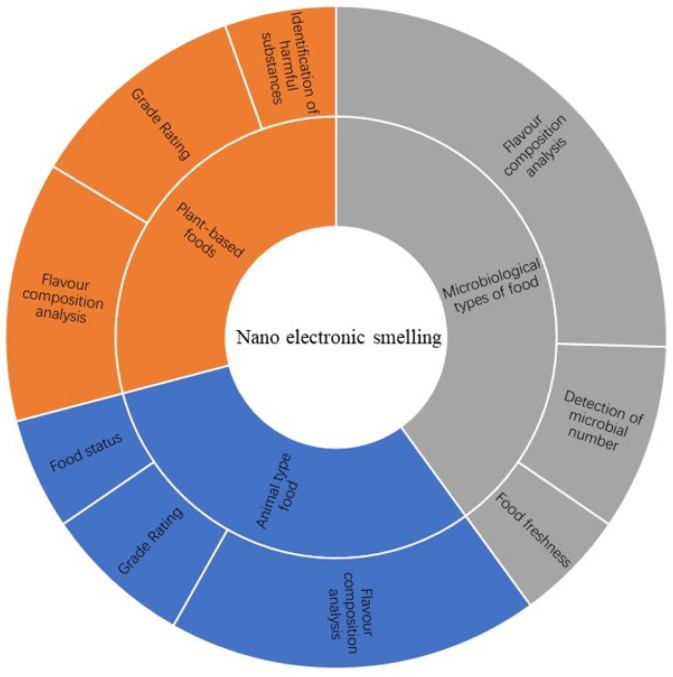
Overview of the distribution of the content.

This paper describes the scheme of the applications of nanoelectronic smelling for different food products in three categories: animal-based food, plant-based food, and microbial food. Table 1 shows the inefficiency of some data. This paper summarizes the application scenarios of nanoelectronic smelling in recent years, as well as its limitations and deficiencies in identifying different types of food. Figure 1 shows the content distribution of the literature reviewed in this paper, in addition to the principles of nanoelectronic smelling and its nanostructure. Finally, we summarize the existing constraints of nanoelectronic smelling and analyze the prospects for future applications to provide ideas for research into nanoelectronic smelling.

## 2. Nanoelectronic Smelling: Analysis of Animal-Based Foods

In animal-based foods, such as chicken, fish, and pork, the state of the meat affects their respective taste and flavor. Nanoelectronic smelling can be an efficient way to distinguish the state of the meat, can be used to evaluate different grades of meat for consumer reference, and can be used to analyze the flavor composition of cooked food such as sausages, braised pork ribs, etc., to provide an effective flavor reference for the producers of these types of food. Table 2 is a summary of the main substances affecting flavor in animal-based foods.

### 2.1. Flavor Composition Analysis of Animal-Based Foods by Nanoelectronic Smelling

Jie Shi et al. [8] proposed the feasibility of applying solid phase microextraction (SPME)-gas chromatography-mass spectrometry (GC-MS) and electronic smelling techniques to differentiate the braised short ribs from different regions. They first analyzed the braised short rib samples for fatty acids, aromatic compounds, and volatile compounds using GC-MS, and discussed that the moisture, protein, and fat contents in the ribs differed greatly between regions, which might be due to the different cooking times in different regions that result in the different moisture loss of the ribs, whereas the difference in protein may be due to the source of the material as well as the cooking conditions. Protein undergoes a Maillard reaction during cooking and oxidizes with fat to produce a meaty flavor, forming the precursors of flavor (free amino acids). Electronic smelling was used to analyze the aroma composition of the samples by W1S, W1W, W2S, W2W, W3C, W3S, and W5C sensors (PEN3 Airsence, Schwerin, Germany), and a total of 183 volatile compounds were analyzed. Hexanal, benzaldehyde, and nonanal were the major compounds found in high concentrations in the braised pork ribs. The only volatile compound found in the Guangdong sample was 4-Isopropylbenzaldehyde, which shows that it is not only the cooking method or the source of ingredients that affects the flavor composition of braised pork ribs, but also the addition of soy sauce, vinegar, ginger, chili, wine, and pepper in different regions, e.g., ethyl acetate could be a volatile component due to the addition of soy sauce or wine, and linalyl acetate is the main volatile component of the leaves and skin of peppercorns.

**Figure 2:**
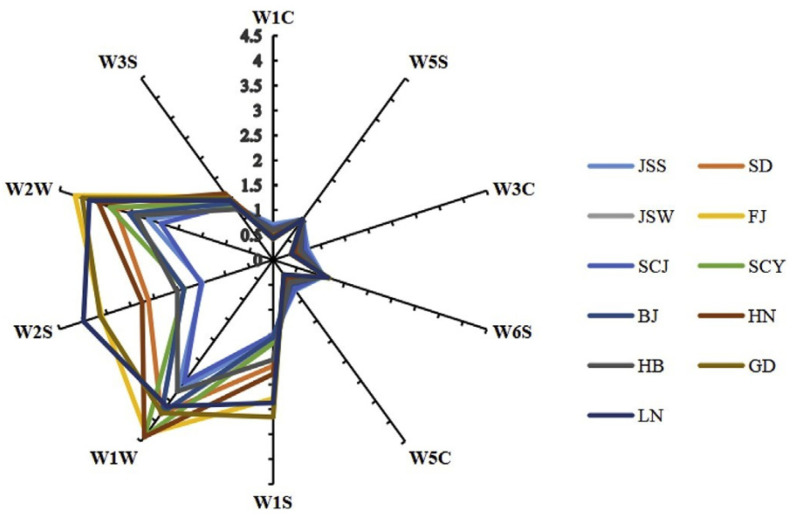
Nanoelectronic smelling mainly uses W1S, W1W, W2S, W2W, W3C, W3S, and W5C sensors. The W1S, W1W, W2S, and W2W sensors had strong and different responses to the aroma components of the samples, indicating that braised ribs may contain high sulfides, terpenoids, alcohols, and aromatic compounds. In the scatter plot, the samples from different regions were well-separated.

Phenolic compounds, acids, furans, and pyrazines were all detected as the flavor components in the braised short rib samples, and highly-enriched volatile components based on the GC-MS analysis were closely associated with electronic smelling signals. As shown in Figure 2, the response of each sensor in the nanoelectronic smelling process to the different flavor components of the sample can be seen, such as W1C, W3C, and W5C sensors, which are sensitive to hydrocarbons and aromatic compounds. In contrast, W1C, W3C, and W5C signals were negatively correlated with the abundance of 1-methyl-4-(1-methylvinyl) cyclohexene, 4-isopropylbenzaldehyde, 1-octene-3-alcohol, and 2-furanol. Combining all the results, hexanal, nonanal, linalyl acetate, 2,5-dimethylpyrazine, and 2,3-octanedione showed a strong correlation with lipids, proteins, and fatty acids. Through the solubilization, encapsulation, and release processes, these key compounds greatly affect the overall aroma of braised short ribs. Nanoelectronic smelling can successfully identify condiments such as soy sauce, vinegar, pepper, rice wine, ginger, and pepper in the samples, supporting the idea that electronic smelling can be used to differentiate braised pork ribs from different regions. The study takes advantage of electronic smelling inside different sensor arrays to identify different flavor substances. This kind of nanoelectronic smelling can be targeted to distinguish a particular kind of substance, which enables researchers to study the foundations of flavor substances.

The desirable flavor of seafood products is one of the main reasons for controlling consumption. Mengyue Hu et al. [17] used electronic smelling and gas chromatography-mass spectrometry to analyze the flavor composition of shrimp from different processing methods. The study used nanoelectronic smelling based on ten metal oxide sensors, as different sensors can respond to different volatile compounds. A total of 48 volatile compounds in five categories of alcohols, aldehydes, nitrogenous compounds, hydrocarbons, and other compounds were detected in the four shrimp samples studied. During testing it was found that the aldehydes and alcohols in shrimp increased sharply after boiling and that a large number of nitrogenous compounds were produced during drying.

The results showed that the concentration of alcohols, as a mild characteristic odor, changed significantly during the different processing methods [18]; volatile alcohols were higher in the boiled shrimp than in the raw shrimp, and 1-octen-3-ol was found to be the dominant alcohol after boiling followed by 2-ethyl-1-hexanol. Aldehydes were not detected in the raw shrimp but five aldehydes were detected in the boiled shrimp: pentanal, hexanal, octanal, E2-heptanol, and benzaldehyde, and eight chain or heterocyclic compounds were detected in the dried shrimp samples, indicating a significant increase in aldehydes during the drying of the shrimp. A total of thirteen hydrocarbons were detected in all shrimp samples, including aliphatic hydrocarbons, branched alkanes, aromatic acids, and olefins. The remaining volatile compounds included sulfur-containing compounds, esters, and ketones, and the results indicated that these compounds did not contribute much to the overall flavor, whereas 1-octen-3-ol with an odor activity value (OAV) value of 28.06 was the strongest odor compound in the boiled shrimp samples. 1-octen-3-ol was identified as a potent aromatically active compound in other culinary seafood with a mushroom odor. In addition, the aldehydes and alcohols had a weaker effect on shrimp flavor and the pyrazines had a stronger effect. Among these compounds, the most effective aromatically active component in dried shrimp was 2-ethyl-5-methylpyrazine, which imparted a pleasant roasted-nut flavor. In addition, 2,5-dimethylpyrazine and 3-ethyl-2,5-dimethylpyrazine also contributed to the dried shrimp flavor. Therefore, in the analysis of the seafood products, pyrazines are the main influencing factors of flavor, so nanoelectronic smelling should pay more attention to pyrazines, followed by alcohols. Alcohols can provide seafood products with other flavors in addition to their own flavor, by enriching the flavor of the products, whereas esters and ketones are less important. This study demonstrates that boiling and post-drying are important stages in improving the flavor quality of shrimp and provides a good direction for processing and production for businesses that produce shrimp.

Nowadays, excessive intake of high-salt foods has become one of the most serious health problems. to reduce the content of sodium chloride in food, In a study of salt-free bovine bone protein extract (NS-BBPE) used to compensate for the salty sensation of healthy low-salt foods, Dongyu Shen et al. [21] optimized the Maillard reaction and were able to detect the salty taste induced by the NS-BBPE odor using nanoelectronic smelling. The study used nanoelectronic smelling equipped with a total of ten metal oxide sensors to analyze the linear discriminant analysis (LDA) of the unsalted beef bone protein extract for its overall level of aroma composition and found high levels of organic sulfides, terpenes, alkane aromatics, and nitrogen oxides in the unsalted beef bone protein extract [22]. This study is similar to Jie Shi et al.’s [8] research method, which further proved such research methods to be very reliable.

This study aimed to prove that salt-free bovine bone protein extract can produce a salty taste similar to that of sodium chloride, using an enzymatic Maillard reaction under the following process conditions: a reaction temperature of 105 °C; a reaction time of 20 min; and a reduction of sugar by 1 g; EH-NS-BBPE by 30 g; water by 10 g; and HVP by 5 g. This finding may provide a replacement for the unpleasant taste caused by excess salt in foods as well as improve the healthiness of food without affecting the taste.

Tilapia meat is an excellent source of protein and Jiahui Chen et al. [19] used nanoelectronic smelling (Alpha M.O.S., Toulouse, France), headspace-SPME-gas chromatography-mass spectrometry, and headspace-gas chromatography-ion monitoring systems to analyze the aroma characteristics of tilapia from four heat treatment methods, i.e., microwave, baking, steaming and boiling, which were used to show sensory differences in raw and cooked tilapia meat. The study used Fox 4000 sensing array fingerprint (4000 sensing array fingerprint) analyzer nanoelectronic smelling based on 18 metal oxide semiconductors. The results showed that tilapia treated by steaming and microwave methods had similar flavor components, that raw and baked fish had significantly different flavor components, and that a total of 43 volatile organic compounds were identified the in raw and heat-treated tilapia. Seven aldehydes, two ketones, five alcohols, one ester, twenty-two hydrocarbons, and six other compounds were identified in the raw tilapia; 7 VOCs (two aldehydes, five hydrocarbons) were identified in the microwaved tilapia; 16 VOCs (two aldehydes, two ketones, three alcohols, seven hydrocarbons, and two other compounds) were identified in the roasted tilapia); 11 VOCs (two aldehydes, one ketone, four hydrocarbons, and four other compounds) were identified in the steamed tilapia; and 13 VOCs (one aldehyde, one ketone, and one alcohol) were identified in the boiled tilapia. These results indicate that volatile compounds can be largely reduced in heat-treated fish. These analyses indicate that tilapia treated by four different thermal processing methods possesses different aroma characteristics, which is essential to understanding the effects of thermal processing on the nutrients in food [20]. From the results, aldehyde, ketone, and alcohol are identified as the main flavoring substances in fish, although the proportion of hydrocarbon is larger only in the boiled fish samples. Studying the references and applications of nanoelectronic smelling for analyzing the main flavoring substances in food also improves study efficiency. This is essential for understanding the effects of thermal processing on the nutrients in food. Furthermore, analyzing the changes in the volatile aroma substances of food by different heat-treatment methods provides a reference for an understanding of the flavor of aquatic-based foods and facilitates the processing and consumption of food products.

The type of wood is the most critical factor affecting smoke composition [9]. Using nanoelectronic smelling, Xiaoyu Yin et al. [10] analyzed the volatile composition of Harbin red sausages smoked using pearwood, oak, applewood, and beechwood chips. A total of 87 volatile compounds, including alcohols, aldehydes, ketones, acids, esters, phenols, terpenes, aromatic hydrocarbons, and sulfur-containing compounds, were detected in the sausages smoked using different methods. Through an analysis of the results, the sausages smoked using pearwood had a higher furfuryl alcohol content than those smoked using oak or beechwood, whereas there was little difference with those smoked using applewood. The applewood-smoked sausages had a higher furfural content than those smoked using oak or beechwood, and it was found that the highest content of 2-hydroxy-3-methyl-2-cyclopenten-1-one was found in the smoked sausage. In addition, 2-cyclopentenone, 2-methyl-2-cyclopenten-1-one, 3-methyl-2-cyclopenten-1-one, 2,3-dimethyl-2-cyclopenten-1-one, 3-ethyl-2-cyclopenten-1-one, and 3-ethyl-2-hydroxy-2-cyclopenten-1-one were also detected, which are common in smoked meats. In the analysis of the individual sensors of nanoelectronic smelling, it was found that the smoking process had a significant effect on the volatile substances of Harbin red sausage; the change in the volatile compounds was the main reason for the enhanced smoky odor, and the smoky odor of sausages smoked using pearwood was more intense. This study provides a reference for distinguishing different types of smoked meat products and provides directions for the type of wood chips that can be used to smoke foods to make them more flavorful.

In the same study of smoked meat products, Lang Zhang et al. [11] investigated the effects of sugar on the flavor characteristics of smoked chicken; the source of its smoked flavor was mainly due to the caramelization of sugar at high temperatures that produces volatile compounds. The pyrolysis products of sugar caramelization differed depending on the smoking temperature and time, so it was important to select the appropriate smoking time used to control the flavor of smoked meat products. In this study, 75 volatile compounds including alcohols, aldehydes, acids, esters, phenols, furans, ketones, and other compounds were detected in the smoked chicken samples by using EN3.5 (PEN3 Airsence, Schwerin, Germany) nanoelectronic smelling. The content of 1-octen-3-ol was found to be the highest among the alcohols and increased with the increase in smoking time. The presence of linalool gives the smoked chicken its fruity and mushroom-like aroma. Other aldehydes include pentanal, hexanal, octanal, nonanal, (pent)2-octenal, furfural, decanal, 5-methylfurfural, and p-anisaldehyde. The odor thresholds of the aldehydes were generally lower than those of the other volatile compounds, and these aldehydes play an important role in the overall odor of smoked chicken legs. Analysis using nanoelectronic smelling revealed that acid compounds contribute little to the flavor of smoked chicken and that among the ester compounds, high levels of vinyl propionate and methyl 2-furancarboxylate were found, which are key to the floral and fruity aroma. Phenolic compounds have a strong influence on the formation of the smoky odor and are mainly derived from cresol and eugenol, and (E)-2-methoxy-4-(prop-1-enyl) phenol, pentane-2-methoxy-4-(prop-1-enyl) phenol along with other phenols give smoked foods a smoky and pungent flavor. Furans are derived from the enolization and dehydration reactions of carbohydrates, and the analysis of 2-acetylfuran, 2-methyl-benzofuran, 5-methyl-2-acetylfuran, and 2-furfuryl-5-methylfuran found that these compounds give the smoked chicken a sweet, fruity, and grassy flavor. Of the nine ketones detected, the levels of 3-octanedione, acetophenone, 4-(5-methyl-2-furanyl) butan-2-one, and 3′-methylacetophenone were high, with 3′-methylacetophenone having the greatest effect on odor, whereas acetophenone gave the smoked chicken a roast coffee odor. The other seven compounds probably came from the spices used in the cooking process and they contributed significantly to the overall flavor. Out of a total of 75 volatile compounds, 18 volatile compounds were identified as the key compounds affecting flavor.

On the whole, the methods used in the nanoelectronic smelling analysis of animal-based food flavors are largely the same, using nanoelectronic smelling sensor arrays to detect different flavor substances. All methods obtained accurate results, which not only reflects the precision and speed of nanoelectronic smelling but also, to a certain extent, reflects that nanoelectronic its robustness. One slight disadvantage is that the different sensor arrays can only analyze specific and already-identified substances, so for substances that are yet to be identified, researchers will not be able to utilize it, which is also a possible direction for the future upgrade of nanoelectronic smelling applications.

### 2.2. Grading of Animal-Based Foods by Nanoelectronic Smelling

The objective evaluation of food is the key to differentiating food grades. Kang Qian et al. [23] developed a portable nanoelectronic smelling system to differentiate three grades of Chinese ham. The nanoelectronic smelling system has a sensing module with a sensor array that works in a similar way to nasal cells to collect volatiles, coupled with a server module and a cloud-stored database that works as a brain nerve to store and analyze the collected signals, and which can be connected to a smartphone for easy control. It broadly contains three parts, sensor chamber, control module, and wireless communication module, each of which can adsorb the target gas and transmit it to the smartphone through the back-end and wireless communication module. The results of the ham analysis by nanoelectronic smelling combined with the feature optimization method, showed that nanoelectronic smelling was able to easily distinguish between three grades of ham and had ideal performance in ham-grade prediction.In the future, the combination of electronic smelling and the feature optimization method could potentially be used to identify other agricultural products. This study combines nanoelectronic smelling with a smartphone, which highlights the convenience of nanoelectronic smelling and paves the way for the future popularity of nanoelectronic smelling.

Yuan Xu et al. [12] studied flavor differences in five different parts of Chinese cut chicken (skin, breast, leg, head, and buttocks) using a nanoelectronic smelling array equipped with 14 sensors, each sensitive to hydrogen sulfide, sulfide, alcohols, ketones, aldehydes, and aromatic compounds, respectively. The analysis of the results revealed 137 volatile compounds, including 21 hydrocarbons, 7 aldehydes, 10 ketones, 53 alcohols, 10 esters, 6 furans, 11 acids, 15 miscellaneous compounds, and 4 sulfur-containing compounds. Twenty-one hydrocarbons were also detected but did not contribute much to the overall flavor due to the high odor threshold, whereas chicken breasts showed a high correlation with volatile compounds such as 2-decenal, the-(C28), 3-heptanone, 5-methyl-(C34), ester acetic acid, amyl ester (C96), propionic acid, hexyl ester (C99), furan 2(3H)-furanone, 5-butyldihydro-(C107), acid caproic acid, 2-ethyl-(C116), dichloromethane (C121), and pyridine, 2-pentyl-(C128). In addition to2-heptanol (C56), 2(3H)-furanone and 5-ethyl-dihydro-(C105) gave the chicken breast a pleasant, nutty aroma with a low aldehyde odor threshold. Compounds such the(E)-2-octenal (C25), hexanal (C23), and 2-pentylfuran (C104) were found to be the most abundant in the chicken breast samples. Aldehydes were found to be the highest in chicken skin compared to others such as (E, E)-2,4-decadienal (C26) and 3-methyl-butyraldehyde (C22), which have the characteristic odor of cooked chicken oil. Benzaldehyde is the key aroma produced by the Strecker reaction and is present only in the head and breast of the chicken. Long-chain and branched-chain alcohols derived from unsaturated fatty acids had a lower odor threshold and were present in greater amounts in chicken skin (which also contained significant amounts of ethanol) but in lower amounts in chicken breast. 1-octen-3-ol (C65), a compound characteristic of meat-flavored fats, was detected only in the thigh and breast samples. Of the furan compounds, 2-ethylfuran (C102), a well-known product of linoleic acid autoxidation, was found only in cooked chicken skin and butter. Ketones, hydrocarbons, esters, and nitrogenous compounds contributed little to the overall flavor due to their low concentrations and high thresholds, whereas hydrogen sulfide and sulfur-containing substances were detected in chicken heads resulting in their poor flavor. Overall, among the five parts of the chicken analyzed for flavor, chicken breast had a better flavor that was acceptable to most consumers, and this study could cater to consumer preferences by improving product quality and could also provide suggestions for different consumer preferences. According to the results, when different parts of the same food are studied using nanoelectronic smelling, specific flavor substances can be classified and identified. For example, if the flavor of chicken skin is studied, the sensor array for identifying aldehydes can be focused on. It is a challenge for nanoelectronic smelling to discriminate between different parts of the chicken, and since there are only a few differences in the flavor substances of the different parts of the same chicken, the analysis becomes much more difficult for nanoelectronic smelling but further proves the accuracy of nanoelectronic smelling methods.

Pork has long been a staple food. Dong Han et al. [13] used nanoelectronic smelling to identify pork from three different breeds of Tibetan, Sanmenxia, and Duroc pigs. An analysis of the results detected 61 volatile compounds including aldehydes, alcohols, ketones, esters, aromatics, hydrocarbons, furans, nitrogenous compounds, and sulfur compounds. Aldehydes were the most abundant in the samples, followed by hydrocarbons and aromatic compounds. The formation of aldehydes from fat through oxidative degradation was the main contributor to the distinctive flavor of cooked pork. Eight aldehydes were detected in the samples, including hexanal, heptanal, nonanal, hexadecanal, 5-2-octthel, (E)-2-nonenal, and two phenyl-containing aldehydes, benzaldehyde, and 4-ethylbenzaldehyde. Hexanal gave the pork a grassy athea and (E)-2-nonenal producd a fatty aroma. A total of seven alcoholic compounds were detected in the samples: the alcohols 1-pentanol, 1-hexanol, and 1-octanol, as well as 1-octen-3-ol, 2-hethedecanol, (E)-2-octen-1-ol, and anisic brain. Among these unstable factors, the compounds 1-octen-3-ol, thectanol, and (E)-2-octen-1-ol were found in all three substances with various boiled pork products. 1-octen-3-ol gave the pork a mushrootheike taste and (E)-2-octen-1-ol produced a green apple-like taste. Among the three furan compounds detected, 2-pentylfuran, which had a fruity and buttery taste, was the most abundant, and the two nitrogenous compounds, pyridine and 2-acetylpyrazine, were significantly higher (*p* < 0.01) in boiled pork from porcine triceps brachii than in boiled pork from porcine biceps femoris. In addition, 3-methylthiophene and benzothiazole were very abundant in boiled meat from porcine biceps femoris. The detected aromatic compounds did not contribute much to the pork flavor due to their high odor threshold. Pine oil acetate and ethyl acetate were only found in Duroc pork and can be used to differentiate between different breeds of pork. Two flavor murmurs, 2-pentyl flavor murmur and 2-ethyl flavor murmur, impart a rubbery and sweet flavor to cooked pork, respectively. From the nanoelectronic smelling analysis, out of the 61 volatile compounds found, 25 of them were pork odor active compounds, and hexanal, nonanal, 1-octen-3-ol, dimethyl disulfide, heptanal, 2-pentyl flavor murmur, and 2-ethylfuran were the main contributors to the overall flavor of boiled pork, with their odor activity values (OAVs) ranging from 17.3 to 524.2 for the three pig breeds. Boiled pork was clearly distinguishable and 12 odor-active compounds including (E, E)-2,4-decadienal, ethyl caproate, dimethyl disulfide, hexanal, 2-acetylthiazole, (E)-2-nonenal, 1-0-octene-3-OI, (E, E)-2,4-nonenal, heptanal, (v)-2-octen-thel, styrene, and (E)-2-octenal were identified as potential flavor markers. Overall, the volatile compounds from different varieties of pork could be well-differentiated by nanoelectronic smelling analysis, which is a potentially viable method for evaluating pork. Similar to the study by Yuan Xu et al. [12], the discrimination of pork breeds provides a good reference for nanoelectronic smelling in distinguishing food grades, sources, and parts.

Zelin Duan et al. [14] used a nanoelectronic smelling analysis of different salmon species to differentiate the flavors of Chinese rainbow trout (ChR), Chilean Atlantic salmon (CA), Chinese Atlantic salmon (ChA), and Chilean rainbow trout (CR). and to study the odor characteristics of the dorsal muscles of salmonids from different geographical origins. An analysis of the results showed that (Z)-4-heptenal, furfural, 2,3-butanediol, 2,3-butanedione, 1-octen-3-ol, and 2-methylbutyraldehyde were higher in Chinese rainbow trout, and that 1-octen-3-ol produced a plant-like aroma and mushroom-like odor of the flesh. 3-furanomethanol and 5-methyl-2-furanomethanol were found to be higher in Chinese rainbow trout and 5-methyl-2-furanomethanol complemented the minty and herbal odor of the fish, producing a pleasant sweetness and characteristic odor. In contrast, (Z)-4-heptanal has a fishy boiled potato flavor and is made from (E, Z)-2,6-nonadienal, which is the primary odor-active compound for many marine organisms such as fish, crabs, and mussels. Heptanal (monomer) has been detected in Chilean rainbow trout but is not found in other species of fish. It is produced due to the oxidation of fatty acids. The unique volatile compound in Chinese Atlantic salmon is 3-methyl-2-butanol. Acetaldehyde gives the fish the aroma of almonds and caramel and the content found in Chinese rainbow trout and Chilean rainbow trout is significantly higher than that found in Chilean Atlantic salmon and Chinese Atlantic salmon. Duan et al. also found that some volatile compounds showed some regularity and could be broadly grouped into two categories, with Chilean rainbow trout, Chinese Atlantic salmon, and Chilean Atlantic salmon in one category and Chinese rainbow trout in the another. High levels of 2-butthene, pen-tanal, and (E)-2-hexenal were found in the first category, probably because these fish are farmed in seawater. In addition, (Z)-4-heptanal, furfural, 2,3-butanediol, 2,3-butanedione, 1-octen-3-ol, and 2-methyl butyraldehyde had significantly higher signal intensities in the second category than in the first category, and these results suggest that the alinity of environmental salinity on salmon flavor may be greater than species differences alone. This study attempts a method for the rapid differentiation of fish species origins using nanoelectronic smelling, which can be effective in accurately distinguishing salmonid fish production regions as well as production conditions from a market perspective, providing a new way of authenticating food safety, and can also be used as a tool for identifying food fraud in the marketplace.

In the nanoelectronic smelling evaluation of food-grade research, all reflect the good accuracy of the nanoelectronic smelling, can be separated from the small material differences, which provides a good reference for the future popularization of the nanoelectronic smelling application, this has paved the way for its popularization, but the slight deficiency is that the nanoelectronic smelling can only make a distinction for specific foods at present, and it will be a good research direction to popularize it in all foods.

Above all, nanoelectronic smelling in hierarchical identification in the field of plant foods, does not appear particularly gratifying advantages, this may be due to poor flavor ingredients in plant-based foods very subtle and easily affected by the external environment, existing nanoelectronic smelling cannot accurately distinguish, but can be combined with the model, find out the specific flavor components, precision criterion, So this can make the classification of food more clear, and the application of nanoelectronic smelling combined with a variety of methods may be the future application trend.

### 2.3. Status of Nanoelectronic Smelling Analysis of Animal-Based Foods 

Jun Qi et al. [15] investigated the effects of different freezing and storage times of fresh chicken meat on its stew flavor by analyzing changes in free amino acids, 5′-nucleotides, minerals, and volatile components. The total free amino acid stew content increased from 159.90 mg/100 mg to 292.81 mg/100 mg as the freezing and storage period of raw meat was extended. A total of 58 volatiles, including aldehydes, ketones, alcohols, hydrocarbons, furans, esters, and sulfur-containing compounds, were detected after the chicken was frozen and stored for 0, 2, 4, 6, and 8 weeks and then stewed for 3 h. The odor-active compounds consisted of 12 aldehydes, 3 alcohols, 2-heptanone, and 2-pentylfuran. These results indicated that aldehydes were the most important aroma substances, and that hydrocarbons and esters did not contribute much to the overall aroma due to the high odor threshold. In this study, since enzymes in meat cannot synthesize mineral elements, changes in the mineral element content could only be related to water and heat transfer, and since the chicken samples were already boned, flavor substances from the bone were transferred to the meat during stewing, thus causing flavor changes in the meat. This could also account for the increase in magnesium and sodium. As the freezing time increased, the content of all mineral elements, except chlorine, decreased, indicating that fewer minerals were transferred from the bone marrow to the meat than from the meat to the broth, results consistent with an increase in the mineral elements in the broth. The increase in aldehydes in the raw meat after 2 weeks of frozen storage could be related to the lipolysis of phospholipids; saturated and monounsaturated fatty acids have high stability, but polyunsaturated fatty acids are unstable. The content of unsaturated free fatty acids in thawed chicken meat increased due to lipolysis of phospholipids. The aroma compounds in the stewed chicken meat are mainly from the oxidative decomposition of polyunsaturated fatty acids. Therefore, phospholipid lipolysis affects the formation of volatile compounds during stewing. The total content of aldehydes decreases and the content of highly hydrophobic substances increases as the frozen storage time of raw meat increases. Short-term storage of frozen ingredients had a positive effect on the improvement in the salty, fresh, meaty, and fatty characteristics of stews, but the grassy characteristics of frozen meat also increased after 8 weeks of storage, similar to raw meat stored at −18 °C for 6 weeks. where subsequent stewing showed improved salty, fresh, meaty, and fatty characteristics. The increase in saltiness and freshness was attributed to the enhanced migration of chloride and fresh amino acids from the bone marrow to the meat. In conclusion, the freezing and preservation time of raw chicken meat can significantly affect its flavor when stewed, with the best flavor and maximum meatiness, freshness, and saltiness of the stew after 6 weeks of freezing. This study provides a good reference for storing frozen fresh meat.

Also analyzing the status of chicken, Esmaeil Mirzaee-Ghaleh et al. [24] investigated the ability of a nanoelectronic smelling machine to identify frozen and frozen-thawed chicken meat, which applied nanoelectronic smelling based on eight metal oxide semiconductors to pretreat the samples. The results showed that the average accuracy of fresh-chilled and frozen-thawed chicken classifications were 95.2% and 94.67%, respectively. The accuracy of the fresh chicken classification was 95.83%, which was higher compared to the frozen-thawed chicken. Nanoelectronic smelling combined with the F-KNN algorithm was used for the intelligent classification of fresh and frozen-thawed chicken meat. The whole system includes sensor signal acquisition, pre-processing, feature extraction, and classification using the F-KNN algorithm. Based on the excellent performance of the F-KNN classification algorithm, it can be concluded that the nanoelectronic smelling system is a fast and nondestructive online identification technology for fresh and frozen-thawed samples. The analysis of the results shows that the high performance of the F-KNN algorithm confirms that the nanoelectronic smelling system can be used as a fast, accurate, and non-destructive method for online and automatic pairwise identification of fresh and frozen-thawed chicken. This study provides an effective identification pathway for the frozen food industry. Using the same analysis of frozen chicken as Jun Qi et al. [15], this study combined nanoelectronic smelling with an F-KNN algorithm to build a complete classification model, which is more reliable and accurate than discriminating solely using nanoelectronic smelling, and also makes a reference for the application prospects of combining nanoelectronic smelling with other methods. In this era of artificial intelligence, combining algorithms to build prediction models may be a new research direction for nanoelectronic smelling.

Fedor S. Fedorov et al. [16] detected the status of chicken differently, using a combination of nanoelectronic smelling and machine vision techniques to check the cooked status of roasted chicken. To measure the environmental changes caused by the presence of volatile compounds, they applied a home-made electronic smelling system that included an array of eight commercial sensors, MQ-2 (smoke), MQ-3 (alcohol), MQ-4 (methane), MQ-5 (LPG), MQ-7 (CO), MQ-8 (H2), MQ-9 (CO, methane, LPG), expressed as CO-II, and MQ-135 (NH_3_, CO_2_, nitrogen oxides). During grilling or cooking, the food undergoes physicochemical changes causing the release of volatile gases and changes in the color of the food. They first cooked chicken with charcoal and after 20–25 min, detected an increase in the sensor response of the nanoelectronic smelling system, possibly related to the carbon dioxide, methane, carbon monoxide, and nitrogen oxide vapors being emitted by the burning charcoal. The beginning of grilling also showed an increase in sensor resistance in the array, which was very pronounced during the first 10 min of cooking. The measured temperature on the surface of the chicken breast was 190–210 °C. At such high temperatures, volatile organic compounds are expected to be formed via the Maillard reaction, thermal degradation of lipids, and Maillard-lipid interactions. The results showed that the primary sources of the chicken’s odor were sulfur-containing compounds including 2-methyl-3-furanethiol, 2-furothiol, and methanethiol; and carbonyl compounds, such as hexanal, trans-2-octenal, and trans-2-nonenal. In particular, MQ-3 and MQ-7 exhibited the highest variability at all cooking times, responding primarily to alcohol vapor and carbon monoxide. The MQ-2 and MQ-4 sensors also exhibited good sensitivity to match the acyclic saturated hydrocarbons. In this study, nanoelectronic smelling was used to evaluate the odor of roasted chicken, machine vision was used to detect changes in appearance, and differences in cooking status were obtained by applying a linear discriminant analysis to the nanoelectronic smelling vector response and Red-green-blue (RGB) data. The production of the roasted chicken odor was controlled by moisture loss during cooking, which released volatile compounds such as aromatic and sulfur-containing compounds. The presence of high concentrations of aerosol particles at 5–10 min was also observed. The appearance of the chicken was also influenced by the grilling time, with an increase in discoloration over time. A linear discriminant analysis separated the clusters associated with "undercooked", "cooked", and "overcooked" chicken. The combination of electronic smelling and computer vision ensured greater selectivity, as evidenced by the increase in the martingale distance between clusters in the LDA plot. Thus, the proposed technique is attractive for food quality control due to its objectivity, rapidity, and non-destructive measurements. Nanoelectronic smelling can identify the odor characteristics of roasted chicken, whereas computer vision can identify the discoloration of chicken meat. The combination of these two methods yields greater selectivity in the qualitative determination of chicken cookability. Unlike MEsmaeil Mirzaee-Ghaleh et al. [24], this study is a novel approach as it identifies the color of cooked chicken using machine vision techniques rather than just algorithms to build a model. This approach can be considered novel, as distinguishing food by appearance is an important criterion for most people, and the use of machine vision technology is a good substitute for human observation. By simulating the basis of human judgment in combination with odor judgment by nanoelectronic smelling, the results of this study become more reliable.

For studying the state of food, researchers are not limited to using only one technique of nanodot electronic smelling, but are able to combine it with algorithms, vision, and other techniques to make it more robust, which could be a future direction for interconnecting nanodot electronic smelling with multiple techniques.

## 3. Analysis of Plant-Based Foods by Nanoelectronic Smelling

Unlike animal-based foods, plant-based foods are not as processed or are processed in a simpler way, such as baking or drying. In this field of research, researchers mostly analyze plant-based food flavor or grade evaluation, such as analyzing the roasted components in soybeans or the aroma of tea leaves after drying. In addition to this, because plant-based foods are closely related to agricultural products, issues such as pesticides, diseases, and other problems can be overcome with the help of nanoelectronic smelling due to its ability to identify harmful substances in food. Table 3 is a summary of the main substances affecting flavor in plant-based foods.

### 3.1. Flavor Analysis of Plant-Based Foods by Nanoelectronic Smelling

Junhua He et al. [25] applied nanoelectronic smelling to analyze the effects of different microwave treatment times on the flavor of tea seed oil and detected volatile compounds. The results showed that the response values of sensor arrays W1W (sulfide), W2W (organic sulfide), and W5S in the nanoelectronic smelling system increased significantly (*p* < 0.05) with increasing microwave treatment times (nitrogen oxides), whereas the response values of W1W, W2W, and W5S were close at 0 min and 2 min but gradually increased from 3 min to 6 min, which showed that microwave treatment increased sulfides, organic sulfides, and nitrogen oxides in tea seed oil. Significant changes in sulfides (e.g., methionine, dimethyl sulfoxide), nitrogen heterocyclic compounds (e.g., pyrazine, pyrrole, and pyridine), and oxygen heterocyclic compounds (e.g., furan, pyran, furanone) were responsible for the significant increase in the WIW, W2W, and W5S response values. A total of 80 volatile compounds were detected across all sensor arrays with similar flavor profiles (green, sour, and fruity) for the samples at 0, 2, and 3 min of treatment time, 4 and 5 min of treatment time for the samples with similar flavor profiles (roasted, fatty), and 6 min of treatment time for the samples in one category (caramelized, roasted). These results provide theoretical guidance for the processing and flavor regulation of oilseed oils. Consumers can choose different flavor types of camellia oil according to their preferences.

Xiaoai Chen et al. [27] studied the changes in the flavor components of bergamot during curing with nanoelectronic smelling. In the treatment, they first ground 3.0 g of sample into a 50 ml vial and equilibrated it for 30 min at room temperature. Then the top space gas was sampled and pumped into the sensor array at a constant rate of 400 mL/min, and the measurement lasted for 120 s. The aroma characteristics of each sample were expressed in terms of the nano response values of the ten sensor arrays of the electronic smelling system. Sensor W2W (aromatic component, sensitive to organic sulfides) contributed to the fresh samples. In other words, the fresh buddha contains more aromatic compounds. Sensors W1S (sensitive to methyl), W2S (sensitive to alcohols, aldehydes, ketones), W6S (selective mainly to hydrides), and W1W (sensitive to sulfides), indicated that the sugary and cooked buddha contains more alcohols, aldehydes, ketones, hydrides, and sulfides. The volatiles of salt-impregnated samples, desalted samples, and sugar-impregnated samples were determined by W1C (aromatic component, benzene) and W5C (short-chain alkane aromatic component) sensors, indicating that salted, desalted, and dried Buddha’s hands contained more aromatic components, benzene, and short-chain alkane aromatic components. According to the results of the nanoelectronic smelling analysis, a total of 81 substances were detected, including terpenes (21), aromatic hydrocarbons (11), alcohols (11), aldehydes (10), esters (7), phenols (6), acids (5), ketones (2), and other species (10). From this analysis, the processes that significantly affected the flavor of bergamot were curing and drying, whereas salting promoted the production of aldehydes, esters, and acids but caused the alcohols to decrease, and drying promoted the production of alcohols, phenols, aldehydes, and acids at the expense of terpenoids. The characteristic volatile compounds during bergamot curing were formed mainly by the biological reactions in the salting stage and the thermochemical transformations in the drying stage. The results show that during the curing process of bergamot, the flavor compounds of different sensor arrays have specific responses and researchers can alter specific samples accordingly, for example, sensor W2W could generate a response to aromatic compounds and organic sulfide, as curing is a state of change, and can then use a real-time monitoring sensor response value to identify changes in the material. This study exemplifies the applicability of nanoelectronic smelling technology in tracking changes in the flavor composition of cured foods, with the advantages being fast, sensitive, and nondestructive sample identification that objectively reflects the information of the sample under test.

Jiashen Cai et al. [28] used nanoelectronic smelling to study the effects of baking methods on the volatile components of soybeans. According to the analysis of the results, there was no significant difference in protein content under low baking temperature or short baking time (*p* < 0.05), because 170 °C or less than 20 min was considered as a mild treatment level. Most of the samples with 10 min baking intervals (except 230 °C) increased the PDI values in soybeans to varying degrees, with roasting at 140 °C for 20 min providing a maximum value of 40.97%. Roasting at 200 °C for 20 min was considered wise for soybean processing, with approximately 21% of the PDI being retained. By increasing roasting temperature, the thermosensitive amino acids were methionine > arginine > cysteine > lysine, with a significant decrease of 12.5–17.7%, followed by serine, histidine, threonine, and tyrosine with a loss of 6.3–12.1%. The content became lower with further increases in baking temperature. The results showed that temperature had an effect on physicochemical indices, except for fat content. Roasting at 200 °C for 20 min decreased the protein dispersion index by about 38%; whereas lipoxygenase and peroxidase were completely inactivated. The major heat-sensitive amino acids were methionine, arginine, and cysteine. The nanoelectronic smelling system showed some ability to discriminate between different roasted soybeans. The study also selected the flavor markers of soybeans to predict the flavor development of soybeans, which is one of the directions for the development of nanoelectronic smelling applications. In summary, roasting at 200 °C for 20 min is considered to be the best method for soybean processing, resulting in an effective inactivation of endogenous enzymes and acceptable nutritional values. The results of this study can be used to gain a preliminary understanding of the relationship between heat treatment and the quality development of soybeans. Further research on the applications of nano electronic smelling to build predictive models for the selection of target volatile compounds, particularly effective flavoring agents, using different soybean varieties and roasting methods is ensured. The research area of nanoelectronic smelling is broadened.

Yanqin Yang et al. [29] applied nanoelectronic smelling to analyze the effects of the drying process on the flavor of black tea, and they used a Herakles II electronic smelling system to output a complex signal instead of the signal generated by a limited number of sensors. It gives a "fingerprint" of volatile components and allows differences or similarities between samples to be demonstrated by powerful data-processing software. In this study, volatile gases were collected from black tea during harvesting, withering, kneading, fermentation, first-drying, and final firing at variable temperatures, and the aroma of black tea was classified into three types: clear, floral, and sweet [35]. Based on the analysis of the results, a total of 243 compounds were identified and some volatile compounds such as (Z)-2-heptenal (82), 1-octen-3-ol (93), heptanal (69), hexanoic acid, and ethyl ester (102) could produce grassy, mushroom-like, and fruity flavors and were present in higher concentrations in clear aroma black tea than other tea samples. Similarly, several specific compounds such as 3-hexenal (42), 3-hexen-1-ol (63), linalool (143), and 2,6-dimethyl-2,6-octadiene (104) may contribute to the specificity of floral black tea samples and they also showed higher levels in floral black tea samples compared to other black teas. The production of the unique aroma of black tea can be regulated by adjusting the drying temperature and time. This study contributes to the quality control and identification of black tea and provides technical support and good theoretical guidance for the targeted processing of black tea. 

A study by Jiayu Chen et al. [30] was similar to that of Yangin Yang et al. [29], which analyzed the aroma of leachates from 44 Dianhong black teas. The two studies were broadly similar in approach but differed in the source of the aroma of the black teas, with 61 volatile compounds identified. Among them, aldehydes were the most common, with 2-methylfuran and linalool being the most important components affecting the mass fraction of tea extracts [31]. The results provide a new technical approach for the quality evaluation and control of tea leachates, and both studies provide comprehensive data support and reference for the comprehensive quality evaluation of tea leaves.

Yan Yang et al. [36] combined nanoelectronic smelling and machine learning techniques to propose a BPNN-based transfer learning framework to build a classification model for identifying different wines and Chinese liquors. They first performed a measurement phase and a rinsing phase. In the measurement phase, the sample gas is drawn into the sensor chamber through the inlet at a rate of 400 mL/min. As the sample absorbs on the metal oxide semiconductor sensor, the conductivity increases and then stabilizes at a constant value as the sensor surface is saturated. In flush mode, this flow rate is regulated to 600 mL/min so that the sample line connected to the inlet is backflushed at 200 mL/min and the analyte is removed from the sensor surface, so the conductivity decreases and then stabilizes at another constant value as the analyte is completely removed. The data points are trained as a model for machine learning and the framework is analyzed to prove that the framework can effectively distinguish wine from Chinese liquor. This study demonstrates that electronic smelling, as a non-destructive device, is able to distinguish between wine and Chinese liquor when selecting the best machine-learning algorithm. It offers broad prospects for the further development of wine and liquor evaluation and process control. This will contribute to the standardization of operational processes and cost reduction for manufacturers, and enhance the protection of consumer rights.

### 3.2. Grade Evaluation of Plant-Based Foods by Nanoelectronic Smelling

Guilherme G. Teixeira et al. [37] used a homemade nanoelectronic smelling to classify and evaluate grades of virgin olive oil, which classified olive oil according to commercial grades of fruitiness intensity (ripe fruitiness or light, medium, and strong green fruitiness). The nanoelectronic smelling of this study integrated several systems, namely a heated sampling unit and a heated multisensor detection array. In contrast to other sampling methods, the study used two commercial silica gel heating blankets to heat the samples and then take the top space gas phase, which undergoes adsorption phenomena with the metal oxides and thus identifies the volatiles. It is worth mentioning that the device also includes a diaphragm vacuum air pump to perform integrated system cleaning between sample analyses, which ensures that the sample is not infected and enhances data reliability. According to the analysis of the results, ripe fruity oils were better differentiated from green fruity light oils. The use of nanoelectronic smelling enables the identification of the different chemical volatile compounds that are responsible for the positive and negative sensory properties typical in olive oils and the satisfactory discrimination of oils based on the perceived primary olfactory sensation as well as their intensity. The olive oil analysis is non-invasive, requires a small number of samples, and is able to provide results in a short time (about 15 min). Although successful preliminary results were reported, further research is needed in the future to improve the spectra of the oils studied and to enhance the validation methods applied, and this research could create greater commercial value by assisting producers in the classification of grades of olive oil.

In order to effectively utilize almond skins and reduce the wastage of resources, Jianti Yao et al. [32] used nanoelectronic smelling to evaluate the taste and flavor of bread with almond slices added. The analysis of the results revealed that substances detected by nanoelectronic smelling, such as ethyl caproate, butyl acetate, and propyl butyrate, were considered to be the source of the bread’s fruity taste, and 3-methyl-butyraldehyde and 3-methyl-thiopropionaldehyde were found to be the source of the bread’s aroma due to their "malty/sweet" characteristics. Similarly, phenylacetaldehyde and benzaldehyde, which were mostly detected in the crumbs, produced "honey/rose" and "almond/caramel" odors, as do maltol and styrene, which also give the bread a sweet, malty, and caramel flavor. Thus, the addition of almond bark gives the bread more flavor. The addition of almond bark to the bread did affect some of the textural properties of the bread during refrigeration, enriching the variety of volatile organic compounds and giving the bread a unique flavor. According to their research, it was found that the negative effects on the bread due to the addition of almond bark could be improved by the ultrasonic treatment of the dough. At the same time, the rich nutrients in almond bark may contribute significantly to the health of bread, and further research on the changes in the nutrient composition during bread making is needed to elucidate the nutritional function of almond bark bread. In conclusion, almond bark can be used as an ingredient in breadmaking, which not only helps to produce bread with long shelf life and unique flavor, but also can make full use of the by-products of almond processing, reduce resource wastage, and environmental pollution, and can be used in the development of new types of bread.

Rice is one of the most important agricultural products in human history; however, the aging process of rice seriously affects its flavor and quality. Jinyong Xu et al. [34] investigated the working principles of the electronic smelling device and its application in the evaluation of rice quality based on variation in aroma characteristics. Volatile organic compounds in rice have unique properties and, therefore, can be used for quality deterioration assessment and identification. In nanoelectronic smelling, volatile organic compounds are adsorbed onto the surface of the sensing material where they react with molecules and produce volatile matter responses leading to changes in the electronic signals. Changes in volatile organic compounds produced during rice aging are then detected based on these signal changes. Finally, appropriate pattern recognition methods (e.g., principal component analysis, partial least-squares, linear discriminant analysis, etc.) and several artificial neural networks (e.g., BP, RBF, LVQ, etc.), were used to evaluate the flavor. The results showed that the substances detected by nanoelectronic smelling included 2-acetyl-1-pyrroline (2-AP), aldehydes, heterocyclic, and alcohols, whereas 2-AP was considered to be the main odorant contributing significantly to the rice aroma, which has a special flavor of nuts and popcorn. In the experiments, the cooling rate was found to have a significant effect on the volatilization of the aroma substances, with higher cooling rates prolonging the retention time, whereas at lower cooling rates, 2-AP had a significant positive effect on sensory evaluation. Therefore, an electronic smelling device can be used to monitor the aging process of rice and accurately assess its quality based on the changes in 2-AP content during storage. In contrast, rice stored at a higher temperature (250 °C) and humidity (70%) showed a significant increase in aldehyde content, leading to a significant decrease in flavor quality. Aldehydes are one of the main volatiles of rice aroma and their concentration is an indicator to monitor the rice aging process. Temperature is an important factor affecting changes in aldehyde content, and high temperatures accelerate the rate of lipid oxidation, leading to poorer rice quality. However, the operating temperature of electronic smelling devices is usually between 200 °C and 400 °C, which has a significant effect on the aldehyde content. The high operating temperature is an obstacle to the application of electronic smelling for quality detection based on changes in aldehyde content during rice storage. This study demonstrates the great potential of nanoelectronic smelling for the rapid qualitative detection of rice, as it has many advantages in conventional analysis. Furthermore, reducing the operating temperature, using suitable pattern recognition methods, and developing new predictive models could be the future trends of nanoelectronic smelling.

In that study, although nanoelectronic smelling showed promising advantages, there are still many shortcomings and most of the ideas are still only at the laboratory stage, for example identifying and quantifying a large group of volatile organic compounds, rather than only a specific one. Therefore, electronic smelling devices can only be used for the preliminary detection and classification of rice quality based on changes in these aroma characteristics (for example, VOCs’ molecules are chemisorbed on the surface of MOS-based sensor arrays) as it is susceptible to high-humidity operating environments, which can lead to a baseline drift, thus affecting the stability of the electronic smelling device. The sensors operate at temperatures between 200 °C and 400 °C, leading to the decomposition of aldehydes. In addition, some of the characteristic compounds decrease significantly with increasing storage time, which requires an increase in the detection limit of the sensor array. In addition, suitable pattern recognition methods were not used to analyze the sensor responses, which directly affected the accuracy of the data analysis. In addition, when the composition of the gas mixture changes, especially when differentiating between different rice varieties, the number of sensors in the array needs to be increased to achieve accurate detection. In addition, the poor repeatability of the electronic smelling device from one year to another is a major limitation for its widespread use in rice quality testing.

Jianxin Song et al. [26] applied different drying methods of nanoelectronic smelling: hot air drying (HAD), heat pump drying (HPD), infrared radiation drying (IRD), vacuum drying (VD), vacuum freeze-drying (VFD), and instantaneous controlled pressure-drop drying (DIC) to analyze the volatile components in red dates. The study used nanoelectronic smelling equipped with 10 metal oxide semiconductors (W1C, W5S, W3C, W6S, W5C, W1S, W1W, W2S, W2W, and W3S) and the results showed that 15, 16, 15, 17, and 26 aroma compounds were detected in RJ, HPD, IRD, VD, VFD, and DIC dried samples, including alcohols, aldehydes, acids, esters, and ketones. In addition, acetic acid, propionic acid, 2-methylbutyric acid, butyric acid, heptanoic acid, valeric acid, capric acid, octanoic acid, and capric acid constituted 90% of the aroma compounds in all samples. The responses were significantly higher for W1W (mainly terpene-sensitive) and W5S (broad response) followed by W2W (sulfide sensitive) with values of 6.08~7.64, 4.1~5.10, and 1.98~2.21. All three were present in RJ, whereas the lowest response values for W1W, W5S, and W2W were found in IRD, HPD, and IRD dried samples, respectively. The other sensors had response values of 0 to 1, indicating a very small response. The vacuum freeze-dried dates showed significantly different aroma characteristics compared to the other dried samples, but the response values of the sensors (W1W and W5S) were close to those of the vacuum freeze-dried samples in the metal oxide semiconductor electronic smelling due to the similarity of the sensors (W1W and W5S) [38]. The vacuum freeze-dried dates had the highest aroma components. When studying the changes in volatile substances, drying methods with the same substance can be classified, and the changes in the drying process can be clearly observed by analyzing the sensor data that responds to the substance, and different operations can also be carried out according to the different state of the sample. This study allows for the discrimination between the different drying methods of dates on the market and their subsequent evaluation and classification.

Tatiane Francielli Vieira et al. [39] applied nanoelectronic smelling to evaluate yerba mate samples from three different states in Brazil, with a detection process similar to that described previously., More characteristically, the study used high-performance liquid chromatography, phytochemicals, in vitro antioxidant activity, visible and near-infrared spectroscopy, colorimetric methods, and nanoelectronic smelling together with chemometric methods, leading to a multi-method exploration of the comparative analysis of yerba mate samples. From the results, it was demonstrated that nanoelectronic smelling has good advantages.

### 3.3. Identification of Harmful Substances in Plant-Based Foods by Nanoelectronic Smelling

Identifying species and detecting early disease in plants is very challenging and difficult to implement for an automated device, and the manual identification process is a lengthy one that requires prior knowledge of the plant itself, such as shape, odor, and texture. M. S. Mustafa et al. [40] developed a system to identify herb species and detect their early disease using computer vision and nanoelectronic smelling. The system focused on the extraction of the odor, shape, color, and texture of herb leaves and a hybrid intelligent system to identify 10 species of herbs with 97% and 96% accuracy, respectively. This study can be a good remedy as a single technique to identify herb species. Despite the high requirements of computer vision for species recognition, it is still difficult to achieve better accuracy when using plant samples such as lemongrass with equal shape and texture. Nanoelectronic smelling solves the problem of consistency in shape and texture based on scent, but its performance in re-identifying herbaceous plant species will be reduced when the leaf scent becomes weak. Therefore, a combination of computer vision, electronic smelling, and pattern recognition would be better for the recognition of herbaceous plant species.

To explore the best method for the quantitative detection of pesticide residues in tea, Alireza Sanaeifar et al. [41] applied electronic smelling and confocal Raman microspectroscopy for the detection of chlorpyrifos concentrations, with complementary data obtained using electronic smelling and confocal Raman microspectroscopy (CRM) sensing techniques. Based on the fact that tea leaves with different pesticide concentrations have different volatile compounds and that the responses generated by nanoelectronic smelling vary, various features of the nanoelectronic smelling sensor array were extracted and combined with partial least-squares (PLS), artificial neural network (ANN), and support vector machine (SVM) methods to build a suitable model system, with a total of 108 variables selected to construct the electronic smelling data vector. This study demonstrated the possibility of a multi-technology fusion system based on electronic smelling and confocal Raman microspectroscopy that could be a beneficial alternative for the rapid and safe control of pesticide residues in tea.

Sara Mostafapour et al. [33] examined harmful substances such as formaldehyde in milk using nanoelectronic smelling based on a novel colorimetric sensor array with a mixture of molybdenum disulfide quantum dots and organic reagents. Each element of the nanoelectronic smelling sensor array consisted of a mixture of molybdenum disulfide quantum dots and organic reagents that functionalize the nanomaterials. This is because molybdenum disulfide quantum dots show specificity and a higher affinity for oxygen-functionalized volatile compounds such as aldehydes and ketones. They used this sensor array first for the classification of eight different aldehydes and ketones based on a linear discriminant analysis. Classification accuracies of 96% and 83% were achieved in the training and prediction phases, respectively. The introduced colorimetric sensor array was then used for the semi-quantitative and quantitative analyses of formaldehyde in milk samples. Because the response of the sensor array to humidity affects its accuracy, reproducibility, and shelf life, this study, unlike other studies, investigated the response of our sensor array to humidity before studying its response to aldehydes and ketones. An analysis of the results shows that a significant advantage of the nano–electron ratio is its very low sensitivity to humidity. In addition, the sensor array can accurately distinguish between formaldehyde levels at permissive levels and elevated levels in milk (with an accuracy of about 95%). Next, it was feasible to combine this sensor array with PLSR as a multivariate calibration method for the direct quantitative measurement of formaldehyde in milk without any sample pretreatment. This method proved to be an effective method for building sensor arrays based on simple quantum dot synthesis and assembly and is expected to be extended to other analytes in a similar manner and with different matrices. An effective solution for milk quality detection is proposed.

## 4. Analysis of Microbial-Based Foods by Nanoelectronic Smelling

The flavor components of fermented foods are complex and variable and there are many factors and uncertainties affecting their flavor components. The variety of fermented foods and the different flavor components produced by microorganisms in different environments, such as shrimp paste with different fermentation times and cheese fermented at different temperatures, present a great challenge to nanoelectronic smelling. Table 4 is a summary of the main substances affecting flavor in microbial-based foods.

### 4.1. Flavor Analysis of Microbial-Based Foods by Nanoelectronic Smelling

Most fermented foods derive their flavor from their degree of aging, but the aging process is very lengthy. Hongbo Li et al. [51] applied a nanoelectronic smelling analysis using physical intervention (ultrasonic field, alternating magnetic field, or a combination of both) to assess the aging process of aged vinegar compared to the flavor of naturally aged vinegar, and the nano-electronic smelling results indicated that the odor changed after the synergistic treatment of ultrasonic and alternating magnetic fields. Some of the sensor results showed values closer to the natural aging odor, and the ultrasonic and alternating magnetic field treatment was able to accelerate the aging process of vinegar. The highest effect of accelerated aging was the combination of ultrasonic and magnetic fields, followed by ultrasonic or magnetic fields alone and the natural process (combination of ultrasonic and magnetic field–magnetic field > ultrasonic, or magnetic field–individual > natural process). This suggests that the combination of ultrasonic and alternating magnetic fields would be a natural remedy to change-accelerate vinegar aging. Xiao Xu et al. [42] applied a nanoelectronic smelling analysis of the fermentation efficiency of multi-stage fermentation using glutinous rice supplemented with Fu brick tea (FGR-FBT) compared to conventional fermentation and showed that the aroma profile was higher in volatile alcohols and alkane compounds and more aromatic. Sulfur-chlorine and olefin compounds were generated, and the difference in the nature of volatile compounds formed in the presence of FBT during fermentation could be attributed to differences in the microbial metabolism. This study may lead to a new line of high-quality fermented foods based on the use of foo brick tea and glutinous rice. Rafael Martínez-García et al. [44] applied nanoelectronic smelling to study the flavor of cava with different ways of yeast inoculation. The study applied nanoelectronic smelling with partial least-squares to model the fermentation time of kava separately. The results showed that lactones, isoprenoids, and furan compounds were temperature-dependent; carboxylic acids were dependent on aging time; whereas methyl esters of fatty acids (MEFAs) and terpenoids were present in yeast form. Alcohols, aldehydes, and isoamyl esters of fatty acids (IEFAs) were temperature and aging-dependent and this study demonstrates that nanoelectronic smelling is a suitable tool for use in the wine industry. Anzi Ding et al. [47] applied nanoelectronic smelling to analyze the flavor analysis of three freshwater fish (bleak, crucian carp, and yellow carp) and fermented fish dew. The results were analyzed and the 71 volatile components were d, 3-methylbutanol, 3-(methyl thiyl) propyl aldehyde, 1-octen-3-ol, phenylacetaldehyde, nonanal, dimethyl trisulfide, decanal, and hexanol Based on the results obtained from this study, further work may focus on the direct regulation of fatty acid metabolic pathways. This study may provide a theoretical basis for the production of high-quality fish sauce products. Xiao Zhang et al. [45] applied a nanoelectronic smelling study to collect 49 samples (13 commercial and 36 traditional soybean pastes) and evaluated the relationship between their flavor differences and odorants. The results showed that the flavor differences in the samples were caused more by the concentration of key aroma substances than by their composition, and that these differences were mainly due to the maturation stage of the traditional soybean paste samples and the heating process of the commercial soybean paste samples. Twenty-three and nineteen odorants were identified as key aroma compounds in the commercial and traditional soybean paste samples, respectively, of which fourteen were identified as key aroma compounds in both types of samples. This phenomenon suggests that the flavor differences in the samples were caused more by the levels of the key aroma compounds than by their composition. Acids (acidic) and esters (fruity) were found to contribute more to the overall aroma, with alcohols (floral and malty), aldehydes (malty), terpenes (floral), and sulfide-containing compounds (similar to cooked potatoes) playing an important role in the flavour of commercial soybean pastes. This study provides ideas for product flavors of industrial soybean paste. Because each flour has unique aromatic properties, it is important to understand it in order to obtain the desired flavor compounds. Danielle Laure Taneyo Saa et al. [52] applied nanoelectronic smelling to study the volatile aspects of different bakery products made using both mature and immature grains and transformed by fermented dough of the genus Lactobacillus. The results show that nanoelectronic smelling can distinguish between doughs composed of two types of flour, and the study verifies that, as a first step in the baking process, a rapid analysis can be performed using nanoelectronic smelling to verify the aromatic compounds of each flour and to control whether the flour is the correct type. Using dough samples, nanoelectronic smelling can be used to separate the different genotypes for maturation and fermentation. This study provides an effective reference for the combination of ingredients and processes of fermented products to produce baked products with higher nutritional value. Shan Li et al. [46] applied nanoelectronic smelling to screen three NSLABs from traditional Kazakh cheese with good proteolytic and autolytic abilities: Pediococcus acidilactici R3-5, Staphylococcus epidermidis R4-2, and Lactobacillus rhamnosus R9-6. A control (no NSLAB) was also included, and four different types of cheese samples were generated and analyzed for volatile compounds at different stages of ripening. The results showed that 48 compounds were detected at the three different stages of cheese ripening, including 8 alcohols, 7 acids, 5 aldehydes, 4 ketones, 12 esters, 8 alkanes, and 3 other compounds. Esters and acids were the important components of the volatile compounds. The variation in the number and levels of volatile compounds reflects the differences in the proteolytic and autolytic capacity between the NSLAB strains and commercial ferments. The study used a nanoelectronic smelling assessment to generate odor fingerprints showing significant differences between the dry cheese, cottage cheese, and control cheese. Significant differences in protein content, free-fatty acid content, and acidity were observed between the two. The shortcoming of this study is that nanoelectronic smelling may not detect the effects of microbial interactions between the NSLAB strain of cheese and the commercial ferments on the overall flavor production, and further studies are needed. Yanyan Lao et al. [53] applied nanoelectronic smelling to investigate the effects of enzymatic digestion and fermentation on the flavor and nutritional quality of fermented chrysalis beverages and analyzed chrysalis fermentation broth (without enzymatic digestion) at different fermentation stages, that is, after 0 h, 12 h, 24 h, 36 h, 48 h, and 60 h. The results showed that the sample fermented for 60 h (after 48 h of lactic acid bacteria fermentation and 12 h of brewer’s yeast fermentation) had the maximum response value. However, the samples fermented for 0-48 h (fermented by lactic acid bacteria only) had smaller response values. This implies that lactic acid bacteria fermentation has little effect on the odor of fermented beverages. The difference in aroma between the samples fermented for between 0 and 48 h was not significant, whereas the difference in aroma between the samples fermented for 60 h and between0 and 48 h was significant, verifying that the chrysalis beverage would have a more prominent flavor after subsequent yeast fermentation. Cuiping Yi et al. [54] applied nanoelectronic smelling to analyze the volatile characteristics of six mixed-culture rice flour fermentations, and the results showed that a total of 110 volatile compounds were detected, among which the flavors of Lactobacillus and Gluconacetobacter were roughly the same. The study helped to determine the selection of strains in rice flour fermentation, which could improve the quality of this type of product. S. Ghosh et al. [55] applied nanoelectronic smelling and a recursive Elman network for the temporal analysis of data generated from the tea fermentation process, with the aim of the early prediction of the optimal fermentation cycle of tea. A total of 81 black tea samples were procured from three different tea gardens and they were analyzed using electronic smelling in this study. The results showed that the model can be used in combination with nanoelectronic smelling for the early prediction of the optimal fermentation time in the tea industry for improving the quality of tea. Wenhui Zhu et al. [49] applied nanoelectronic smelling to study the volatile components in shrimp paste fermented for three years and the effects of different fermentation and storage times on the overall aroma characteristics to determine the appropriate fermentation and storage times to ensure the flavor quality of shrimp paste. The results showed that fermentation time had significant effects on nitrogen, inorganic sulfur compounds, and the aromatic components of alkanes and alcohols in shrimp paste, but not on hydrides, alkanes, and organic sulfides. The shrimp paste was fermented for 2 years with good flavor and overall quality. The longer the fermentation time, the more likely the shrimp paste to develop unpleasant flavors, thereby reducing its quality. Cuiping Yi et al. [48] applied nanoelectronic smelling to study the volatile composition of fresh rice flour (FRN) fermented from pure cultures and five commercially available mixed cultures. The results showed that the main volatile compounds of FRN from pure cultures included aldehydes represented by nonanal, octanal, and 2,4-pentadienal, and alcohols represented by hexanol and 1-nonanol. The aromas showed significant changes in the storage time between 0 and 30 h, indicating a decrease in aldehydes and an increase in alcohols and isoamyl alcohols, with the FRN produced by pure fermentation scoring the highest in sensory evaluation, showing a more satisfactory flavor than that of mixed-culture fermentation. Hassan Rahimzadeh et al. [56] applied nano-electronic smelling to analyze the aroma changes during the storage of aromatic and non-aromatic rice, and the results showed that nanoelectronic smelling can be a good assessment of the rice aging process as well as an auxiliary technique to control this process. In a study by Jie Zhang et al. [57], the results showed that the addition of BGL0224 prior to alcoholic fermentation significantly improved the "aroma index" of Cabernet Sauvignon wines, and BGL0224 enriched the variety of volatile aroma compounds in the wines. BGL0224 enriched the variety of volatile aroma compounds in wine and significantly increased the concentration of some aroma compounds such as MCFAEEs, LCFAAEs, and terpenes, which provides an applicable method for the aroma regulation of Cabernet Sauvignon wines. For food microorganism fermentation, the fermentation material in the process of change is particularly important. This allows researchers to improve the application of nano-electronic smelling for analysis for the period of fermentation to target a specific material using nanoelectronic smelling trace analysis.

### 4.2. Analysis of Nanoelectronic Smelling for Fresh Foods

Freeze-drying processes are the most effective preservatives without compromising the appearance of the final product. Pasquale Giungato et al. [58] applied nanoelectronic smelling to analyze the freeze-drying process to extend the shelf life of sea fennel, and this study tested three possible processes to maintain flavor and extend shelf life: drying at 40 °C, drying at 60 °C, and freeze-drying. Color measurements showed that the samples darkened as the drying temperature increased from 40 °C to 60 °C, whereas freeze-drying preserved the initial appearance of the fresh samples. The weight loss remained almost constant for all samples (about 85% of initial weight) but the average water activity was higher in the case of the air-dried samples at 40 °C and low enough in all cases to prevent mold growth. Juan C. Rodriguez Gamboa et al. [59] developed a thin-film semiconductor (SnO_2_) sensor and a portable, compact self-developed nanoelectronic smelling application trained using a deep multilayer perceptron (MLP) neural network to analyze wine spoilage. This study allows the early detection of wine spoilage thresholds to be performed in routine tasks of wine quality control, and the study was also compared to conventional analytical methods of nanoelectronic smelling. The results showed that the degree of spoilage of the three wines could be classified within 2.7 s of gas injection, which means that the method is 63 times faster than the results obtained by the conventional method in the experimental setup. Xuhui Huang et al. [43] applied nanoelectronic smelling to distinguish the odor of fresh and grilled eel, and a total of 155 volatile compounds were detected in both the eels, showing that the main characteristic volatiles of grilled eel were methyl propyl disulfide, dimethyl trisulfide, heptane, octane, and alkene. The grilled eel produced more aromatic compounds, broad-bodied alcohols, nitrous oxide, mushroom alkenes, sulfur-containing organic compounds, and organic sulfides than the fresh eel. The proportions of aromatic compounds, broad-bodied alcohols, nitrous oxides, terpenes, sulfur-containing organic compounds, and organic sulfides were significantly different between the fresh and the grilled eel. For the fresh eel, aromatics compounds, ammonia, broad-alcohols, nitrous oxides, terpenes, sulfur-containing organic compounds, and organic sulfides occupied a proportion of 9%, 10%, 12%, 11%, 8%, and 5%, respectively, whereas the proportion of homologous compounds for the grilled eel were 23%, 4%, 18%,13%, 9%, and 9%. This study demonstrated that nanoelectronic smelling could distinguish between the two odor differences.

### 4.3. Analytical Detection of Food Microbial Counts by Nanoelectronic Smelling

Robert Rusinek et al. [60] applied nanoelectronic smelling to test the suitability of wheat bread for consumption after days of storage and showed that nanoelectronic smelling is a rapid and non-invasive tool for assessing the suitability of bread for immediate consumption after baking. The device detects a loss of aroma during ageing and indicates the time taken for microflora lesions to appear in bread stored under quasi-anaerobic conditions. In turn, an analysis of the volatile compounds emitted by bread stored under quasi-anaerobic conditions showed that the response by the sensor arrays of nanoelectronic smelling was positively or negatively correlated with the number and percentage of the volatile groups in the general odor. This study demonstrates that nanoelectronic smelling can be a good tool for diagnosing microbial counts in commerical breads after baking. Shuang Gu et al. [50] applied nanoelectronic smelling to detect the number of Aspergillus species of rice and showed that the changes in the volatiles (e.g., n-octanol and tetradecane) and several aromatic compounds produced during storage in rice grains after fungal infestation were closely correlated with the fungal species and the total amount as well as the sensor responses of the electronic smelling. The shortcoming of this study was that it was not possible to identify different fungi of the same genus and in the future, the establishment of a large number of different fungi for the same genus of growth and taxonomic models to identify the species and growth stages of unknown fungi by comparing them with standard models of known fungi is a possible future research direction. Unlike this study, Shubhangi Srivastava et al. [61] applied nanoelectronic smelling and a fuzzy controller for the detection of rice strains with rice mosaic disease and their results showed equal significance. Sudipta Hazarika et al. [62] applied a system for the detection of a pathogen called citrus decline virus (CTV) in citrus spp. using nanoelectronic smelling. The study collected the leaves of 62 plant species and detected viral infections using gold standard polymerase chain reaction. The study successfully classified healthy plants and those affected by CTV-induced disease. The results showed that the system had shorter response times (3 min) and recovery times (10 min) for rapid and largescale screening in plantations and nurseries, and compared to traditional methods, this technique is cheaper, simpler, and saves time. The shortcoming of this study was that the variation in the nanoelectronic smelling performance due to environmental fluctuations was not investigated, which may lead to bias in practical applications. Xiaoxu Zhang et al. [63] applied nanoelectronic smelling to the identification and prediction of three aspergillus ochratoxin and detected a total of 50 volatile compounds. Combined with a partial least-squares regression model, nanoelectronic smelling proved to be a reliable identification and diagnosis method for predicting aspergillus strains on grape culture media. Table 5 shows a comparison of nanoelectronic smelling combined with various methods.

## 5. Conclusions and Outlook

This paper reviews the applications of nanoelectronic smelling in three types of food and discusses the research progress of nanoelectronic smelling in terms of flavor composition analysis, grade evaluation, etc. The substances most identified were alcohols and aldehydes, and it was also shown that they are the factors that influence the flavor of foods. Aldehyde compounds in animal-based foods had the greatest influence on flavor, followed by alcohols on the flavors of plant-based and microbial-based foods. Esters and aldehydes also have a certain influence, as do sulfides and aromatic compounds in animal- and microbial-based foods. Nano electronic smelling will become a hot research direction in the field of nanosensors because of their excellent accuracy and good selectivity.

With advancement in microelectronics, material sciences, manufacturing processes, and computer technologies, nanoelectronic smelling devices are developing toward integration, miniaturization, and practicality. Combining nanelectronic smelling with different algorithmic models from different research objects to form a complete detection and analysis system is the future application trend of nanoelectronic smelling. For example, combining it with a partial least-squares regression algorithm to perform volatile compound measurements. In addition, improving the response characteristics of the sensor array to cope with the impact of different working environmental factors is also a future problem that needs to be solved. Many nanosensor arrays are highly susceptible to environmental factors such as humidity, temperature, etc., which could lead to a baseline drift affecting the stability of the device; however, methods such as pattern recognition can also be used to improve the accuracy of the sensor responses to ensure the reliability of the data. At the same time, how to accurately distinguish between different types of foods belonging to the same genus is another issue that needs to be addressed. Foods belonging to different species have high similarity in flavor components, therefore, higher precision requirements for sensors are required, which greatly increases the application limitations of nanoelectronic smelling. The density of the sensor arrays could be increased to break this limitation, and one solution could be to start with nanostructures or nanomaterials. In the near future, nanoelectronic smelling could not only be used in food identification, such as disease identification, environmental monitoring, etc., but it could also be developed into smaller and more professional equipment so that it could be more widely used and could even replace many traditional time-consuming industrial processes. Further progress needs to be made in the field of nanoelectronic smelling-based sensors, and the range of applications can be further expanded.

## Figures and Tables

**Table 1:** Efficiency and inadequacy of existing data.

Insufficient Data Available	Reference
Sampling too little data, if there is an emergency, errors may occur	[4]
Specific sensor arrays can be designed for specific flavor components	[6]
For large data analysis, experiments can be combined with principal component analysis, partial least-squares method, and other methods to establish a specific model for prediction	[7]

**Table 2:** Summary of main parameters of animal-based foods.

The Main Ingredients That Influence Flavor	Reference
Aldehyde	[8,9,10,11,12,13,14,15,16]
Alcohols	[12,13,14,17,18]
Ketones	[11,14,19,20]
Esters	[11]
Phenolic	[11]
Furan	[11,12,13]
Sulfide	[12,13,16]

**Table 3:** Summary of main parameters of plant-based food.

The Main Ingredients That Influence Flavor	Reference
Sulfide	[25,26]
Aromatic compound	[27]
Benzene	[27]
Acids	[28,26]
Aldehyde	[29,30,31,32,33]
Esters	[29,32]
Furan	[30,31]
Alcohols	[30,31]
2-AP	[34]
Ketones	[33]

**Table 4:** Summary of main parameters of microbial-based foods.

The Main Ingredients That Influence Flavor	Reference
Alcohol	[42]
Aromatic compound	[42,43]
Esters	[43,44,45,46]
Aldehyde	[44,45,47,48]
Alcohols	[43,44,45,47,48,49,50]
Sulfide	[43,47,49]
Acids	[45,46]

**Table 5:** Advantages of combining nanoelectronic smelling with other methods are compared.

Combined Approach	Role	Performance	Reference
Combined with SPME-Gas Chromatography-Mass Spectrometry	Comparison with the results of nanoelectronic smelling analysis to verify the reliability of the data	The two methods were compared with each other to produce more accurate results	[8]
Integration with smartphones	Portable nanoelectronic smelling with smartphone	Simple and convenient, easy to operate, and can collect data analysis and processing, availability is strong	[23]
Combined with the F-KNN algorithm	The method builds a complete classification prediction model with more accurate and reliable results	This method can be used as a quick and non-destructive way to separate the status of chicken	[24]
Combined with machine vision technology	Visual analysis by color change combined with odor analysis by electronic smelling	Analysis from both appearance and odor of food, one more dimension than traditional method, accurate results	[16]
Combining methods such as partial least-squares (PLS), artificial neural networks (ANN), and support vector machines (SVM)	Development of a complete mathematical model for predicting pesticide residues in tea	The complete mathematical model system can be applied in a variety of occasions anytime and anywhere, without environmental restrictions	[41]
Combining deep multilayer perceptron (MLP) neural network training	Applying machine-learning techniques to train and form predictive models from datasets collected by nanoelectronic smelling	Early predictions can be made in the quality control of wine for subsequent changes	[59]

## Data Availability

Not applicable.
